# Influenza A Virus PB1-F2 Gene

**DOI:** 10.3201/eid1210.060511

**Published:** 2006-10

**Authors:** Roland Zell, Andi Krumbholz, Peter Wutzler

**Affiliations:** * Friedrich Schiller University, Jena, Germany

**Keywords:** Influenza A virus, PB1-F2, proapoptotic protein, letter

**To the Editor:** Recently, Chen and co-workers described the expression of an 11th influenza A virus protein, designated PB1-F2 because this protein is encoded in the +1 open reading frame of the segment-2 RNA (*1*). Later, Chen et al. presented a preliminary analysis of 336 PB1 sequences from GenBank (*2*). We have extended the work on PB1-F2 and analyzed 1,864 partial and complete segment-2 sequences deposited in GenBank; these sequences belong to 79 influenza A virus subtypes. In summary, the following 8 observations should receive attention:

First, the size of PB1-F2 polypeptides ranges from 79 to 101 amino acids (aa); most isolates encode versions of either 87 or 90 aa. Because polypeptides of 79 aa are located within mitochondria, their truncation has no effect on the protein function. The frequency of the 79-aa PB1-F2 is ≈5%.

Second, a functional PB1-F2 is expressed by 92% of all segment-2 sequences, i.e., a polypeptide >78 aa. The proportion of intact PB1-F2 varies according to host (humans 90%, swine 76%, other mammals 100%, birds 95%).

Third, the H1N1 subtype comprises 3 genetic lineages. One clade has 2 branches: 1 branch includes the human viruses, with the pandemic 1918 virus at its root; the other branch includes the classic swine viruses. The third clade represents the European porcine isolates. Although all classic swine sequences have a truncated PB1-F2 (in-frame stop codons after 11, 24, and 35 codons), the early human isolates (H1N1 sequences from 1918 through 1947) have an intact PB1-F2. After 1956, however, a mutation became prevalent such that the recent sequences starting from A/Beijing/1/56 terminate after 57 codons. An exception to this rule is A/Taiwan/3355/97. Two human H1N1 isolates with an intact PB1-F2 coding sequence cluster in the H3N2 clade (A/Kiev/59/79, A/Wisconsin/10/98). The PB1 sequences of European porcine influenza A virus isolates cluster with European porcine H3N2 and H1N2.

Fourth, all H2N2 sequences are monophyletic and encode an intact PB1-F2. Fifth, the main sequence cluster of the H3N2 subtype comprises 3 branches: 1) porcine H3N2 and porcine H1N2 sequences from the United States, 2) porcine H3N2 isolates from Hong Kong and human H1N2, and 3) recent human H3N2 and some Japanese H3N2 isolates. Most of these sequences encode an intact PB1-F2.

Sixth, the cluster of European porcine influenza A virus isolates comprises the subtypes H1N1, H1N2, and H3N2. The lack of distinct clades for each subtype indicates frequent reassortment in the evolution of these viruses. Of the segment-2 sequences, 56% encode an intact PB1-F2.

Seventh, other porcine isolates of various subtypes represent transspecies infections or single reassortment events. And eighth, the segment-2 sequences of many avian influenza A virus isolates encode intact PB1-F2. Considerable proportions of truncated PB1-F2 genes were found in the H5N2, H6N6, H9N2, and H13N2 subtypes. However, because of the small number of sequences available, this observation may not be important.

In conclusion, PB1-F2 is expressed in most avian and many porcine influenza A virus isolates. This finding contrasts with those in the initial publication, which stated that PB1-F2 is not expressed in many animal isolates, particularly those of porcine origin (*1*). Because PB1-F2 was described as a proapoptotic protein probably counteracting the host immune response, why numerous human and porcine isolates lack this protein without selective disadvantage remains unclear.
