# Supplementary material for: Influenza A Virus PB1-F2 Gene
Source: Emerg Infect Dis. 2006 Oct;12(10):1607–9. doi: 10.3201/eid1210.060511 (PMC3413943; doi:10.3201/eid1210.060511)
Supplement: Related texts

# Reply

**DOI:** 10.3201/eid1210.060909

**Authors:** Guang-Wu Chen, Shin-Ru Shih

**Affiliations:** * Chang Gung University, Taoyuan, Taiwan

**In response:** Zell et al. (*1*) performed an extensive genetic investigation of PB1-F2, based on up-to-date GenBank sequences. Their sample size (1,864) greatly outnumbered ours (336) in a previous study (*2*) and thus definitely better portrays the genetic characteristics of PB1-F2. We appreciate their analyzing these samples by subdividing nonhuman strains into different species, which we did not do (*2*). Their analysis is especially meaningful for the global pandemic threat from avian influenza viruses, which increases the need to study interspecies adaptation and transmission.

Zell et al. found that 92% of PB1 RNA encodes a functional PB1-F2, compared with our 79% (264/334), which supports the increasingly crucial role of PB1-F2 in influenza virology. They found the proportion of intact human PB1-F2 to be 90%, a substantial boost from our 68% (67/99), which was based on data from late 2003 (*2*). This increase is apparently caused by the increasing number of human H3N2 sequences (mostly encoding an intact PB1-F2 compared with H1N1) deposited in the past 2 years.

Human H1N1 from 1918 through 1947 contains full-length PB1-F2, whereas human H1N1 beginning in 1956 has a truncated PB1-F2 after codon 57. As reported by Zell et al., only 3 human H1N1 strains contain full-length PB1-F2: A/Kiev/59/79, A/Taiwan/3355/97, and A/Wisconsin/10/98. The PB1 genes of A/Kiev/59/79 and A/Wisconson/10/98 were found clustered with human H3N2 as a result of natural reassortment between human H1N1 and H3N2 strains. On the other hand, the asynonymous mutation found on A/Taiwan/3355/97 enabled the translation to get past the usual stop codon at position 58, which other H1N1 strains exhibit. A/Taiwan/3355/97 (H1N1) was isolated from a patient with severe pneumonia. Animal study has demonstrated that the existence of full-length PB1-F2 contributed to pathogenesis in mice (*3*). We speculate that the expression of a full-length PB1-F2 may contribute to disease severity in humans.

The C-terminal domain of PB1-F2 contains the mitochondrial signal and can trigger apoptosis in specific immune-related cells. Our recent work (*4*) comparing avian and human influenza A viruses also found that many species-associated amino acid signatures are located on the C terminal of PB1-F2. This finding highlights the importance of further investigating the role of PB1-F2 on interspecies infection.

## References

[R-2-1] Zell R, Krumbholz A, Wulzler P. Influenza A virus PB1–F2 gene [letter]. Emerg Infect Dis. 2006;12:1607–8.1717658710.3201/eid1210.060511PMC3413943
